# Exploring Interactive Survivorship Care Plans to Support Breast Cancer Survivors: Protocol for a Randomized Controlled Trial

**DOI:** 10.2196/23414

**Published:** 2020-12-04

**Authors:** Akshat Kapoor, Priya Nambisan

**Affiliations:** 1 Consumer Health Informatics Lab Department of Health Services and Information Management East Carolina University Greenville, NC United States; 2 Department of Health Informatics and Administration University of Wisconsin-Milwaukee Milwaukee, WI United States

**Keywords:** breast cancer, cancer survivorship, self-management, patient education

## Abstract

**Background:**

Breast cancer is the most common form of cancer among American women, accounting for 23% of all cancer survivors nationally. Yet, the availability of adequate resources and tools for supporting breast cancer survivors has not kept up with the rapid advancement in treatment options, resulting in unmet supportive care needs, particularly among low-income and minority populations. This study explores an alternative means of delivering breast cancer survivorship care plans (SCPs), with the aim of improving survivor morbidity, patient knowledge, and self-management of treatment-related symptoms, as well as addressing inconsistencies in follow-up care visits.

**Objective:**

The overall goal of this study is to improve the uptake of SCP recommendations via an educational intervention for breast cancer survivors, to improve treatment-related morbidity, patient knowledge, self-management, and adherence to follow-up visits. The specific aims of the study are to (1) evaluate the feasibility of the online SCP, and (2) assess the impact of the online SCP on survivorship outcomes.

**Methods:**

We will enroll 50 breast cancer survivors who have completed initial breast cancer treatment into a 2-armed, randomized, waitlist-controlled pilot trial, and collect data at baseline and 6 months. For the first aim, we will use mixed methods, including surveys and personal interviews among the intervention group, to determine the feasibility of providing an online, interactive SCP (called ACESO) based on the survivors’ online user experience and their short-term adoption. For the secondary aim, we will compare the 2 groups to assess the primary outcomes of survivor knowledge, self-efficacy for self-management, perceived peer support, and adherence to SCP-recommended posttreatment follow-up visits to oncology and primary care; and the secondary outcomes of treatment-related morbidity (body weight, fatigue, depression, anxiety, sexual function, distress, and sleep quality). We assess these outcomes by using measurements from validated instruments with robust psychometric properties.

**Results:**

We have developed and refined the online breast cancer survivorship plan, ACESO, with consultation from breast cancer oncologists, nurses, and survivors. Approval for the study protocol has been obtained from the Institutional Review Board. An advisory board has also been established to provide oversight and recommendations on the conduct of the study. The study will be completed over a period of 2 years.

**Conclusions:**

The results of this pilot study will inform the feasibility and design of a larger-scale pragmatic trial to evaluate the impact of an online breast cancer SCP on treatment-related morbidity and self-efficacy for self-management.

**International Registered Report Identifier (IRRID):**

PRR1-10.2196/23414

## Introduction

Breast cancer is the most common form of cancer among American women, accounting for 23% of all survivors nationally [1]. Owing to improvements in advanced screening and cancer therapies, breast cancer patients are experiencing better prognosis and higher survival rates than ever before. Yet, the availability of adequate resources and tools for supporting breast cancer survivors has not kept up with the rapid advancement in treatment options, resulting in unmet supportive care needs [2,3]. A survivorship care plan (SCP) [4], an integral element of cancer survivorship planning, is a document provided to patients completing initial cancer treatment that contains a summary of treatments, a schedule of recommended follow-up visits, and general information on treatment-related side effects. However, for breast cancer survivors, SCPs in their current form, as a static paper document, provide little-to-no benefit over standard patient discharge procedures [5]. Furthermore, despite the inclusion of SCPs in standard oncology practice, the lack of evidence supporting improved health outcomes [6,7] may be attributed to them being passive and generic in nature, as well as their reliance on the patient to proactively check, analyze, interpret, and retain the information they contain on a routine basis [8-10]. Moreover, there is limited research studying the role of eHealth literacy among survivors. Survivors are able and willing to use online computer applications that generate customized survivorship information [11]; however, prior research shows that eHealth literacy is associated with access to digital resources and level of education [12]. Recommendations by the National Cancer Institute (NCI), the American Cancer Society (ACS), and the Institute of Medicine (IOM) [13] have all emphasized the need to evaluate SCPs and explore new delivery models. Therefore, examining alternative means of delivering SCPs to improve both survivor and provider outcomes warrants further investigation.

With consultation from experts, including survivors and providers, we designed and developed a prototype of an interactive and personalized online breast cancer survivorship tool called ACESO (After-Cancer Education and Support Operations). ACESO aims to enhance the continuity of care for breast cancer survivors and improve their follow-up experiences, especially as they deal with posttreatment challenges such as comorbidities and side effects of treatment [14]. By transforming the conventional, paper-based SCP into a personalized and interactive survivorship resource, ACESO enables 2-way interaction with the SCP by allowing patients to use the built-in tool to track survivor symptoms and quality-of-life observations, and in return, to receive timely and customized educational alerts and individual reminders for follow-up visits. Moreover, it provides an online platform for breast cancer survivors to connect and interact with peers on survivorship topics and develop a peer support network.

This study’s overall goal is to improve the uptake of SCP recommendations via an educational intervention for breast cancer survivors, with the aim of improving treatment-related morbidity, patient knowledge, self-management, and adherence to follow-up visits. The specific aims are, firstly, to assess ACESO’s feasibility. Based on results from our preliminary study [14], breast cancer survivors reported high usability and perceived usefulness of ACESO. We will use mixed methods, including surveys and personal interviews, to determine the feasibility of providing an online, interactive SCP (ACESO) based on the online user experiences and short-term adoption of 25 participants. Secondly, we aim to evaluate the extent of ACESO’s impact on breast cancer survivorship. We will enroll 50 breast cancer survivors who have completed initial breast cancer treatment (radiation, chemotherapy, or surgery) into a 2-armed, randomized, waitlist-controlled pilot trial, with SCP only as the control, and ACESO with SCP as the intervention. The primary outcomes will be (1) survivor knowledge, (2) self-efficacy for self-management, (3) perceived peer support, and (4) adherence to SCP-recommended posttreatment follow-up visits to oncology and primary care; the secondary outcome will be treatment-related morbidity (body weight, fatigue, depression, anxiety, sexual function, distress, and sleep quality). Outcome measurements will be collected at baseline and at 6 months. We hypothesize that ACESO users will show greater improvement across the primary and secondary outcomes than the waitlist control group.

## Methods

### Study Design

We will utilize a mixed-methods approach to evaluate the survivor experience and the impact of an interactive SCP tool in this 2-armed, randomized, waitlist-controlled pilot trial using a pretest and posttest design. Study design and reporting will be in accordance with the Consolidated Standards of Reporting Trials (CONSORT) eHealth checklist. We will randomly allocate breast cancer survivors to either the ACESO (intervention) or the waitlist control group. We will measure study outcomes at baseline and at 6 months.

### Sample Size

We will seek to enroll 50 breast cancer survivors into the study; 25 will be randomly assigned to the waitlist control group and 25 will be randomly assigned to the intervention (ACESO) group. When a paired *t* test with a significance level of 0.05 is used, our sample size is estimated to achieve over 85% power in detecting an increment of 5.97 (9.70%) in self-efficacy for self-management scores in the intervention group. Our calculations are based on prior studies evaluating the impact of online interventions on self-efficacy for self-management among cancer patients [15,16]. Allowing for a 10% attrition rate, we estimate that we will obtain complete data on 22 participants in each group.

### Recruitment and Setting

We will recruit 50 patients completing primary invasive breast cancer treatment (radiation, chemotherapy, or surgery) from a breast cancer surgical oncology clinic located in the Southeastern United States to participate in this proposed study. The clinic served approximately 1160 patients in 2019; therefore, it will provide access to a large and diverse pool of prospective participants for successful recruitment. To be included, patients must (1) be diagnosed with breast cancer at the age of 21 years or older; (2) be within 3 months of completing initial treatment for localized breast cancer (ie, total or partial mastectomy, radiotherapy, or chemotherapy; patients still undergoing hormone therapy will be eligible to participate); (3) not have a history of any other form of cancer; and (4) have the ability to read and write at eighth-grade level English. There are no inclusion or exclusion criteria that are based on gender or race; however, we anticipate the recruitment of mostly (n=50) female breast cancer survivors. Initial contact takes place at the clinic, where the patient population is predominantly female and reflective of the racial and ethnic diversity of the county where the clinic is located; this is demonstrated in our expected enrollment table (Multimedia Appendix 1). Certain groups, including Native Hawaiian and American Indian groups, account for less than 1% of the population (per 2018 census data) of the county where the clinic is located; as such, every effort will be made to include participants from these groups, but given the relatively smaller sample size (n=50) of this feasibility pilot study, it is not deemed likely.

### Study Procedure

Our anticipated study timeline is presented in Multimedia Appendix 2. An informational flyer containing a description and contact information for the research study will be enclosed with the SCP provided to each patient at the clinic. The study coordinator will meet with interested patients after the completion of their SCP clinic visit. Prospective participants will be screened to verify that they meet the inclusion criteria. Eligible patients will be provided with detailed study information, and informed consent will be obtained. Enrolled participants will immediately complete an online, self-administered survey using a provided web-enabled device to collect baseline measurements before randomization. To ensure accuracy, the nurse-trained study coordinator will assist all participants in completing the final section of the survey using their conventional SCP to enter details regarding their breast cancer diagnosis, treatment history, and schedule of SCP-recommended follow-up visits. All participants will be emailed a link to complete the online postintervention survey 6 months from the enrollment date. All surveys will be administered using REDCap (Research Electronic Data Capture) [17]. The study protocol has been approved by the Institutional Review Board and will be registered at clinicaltrials.gov prior to commencing the study activities.

### Randomization

Upon obtaining baseline measures, the biostatistician will use SAS software (version 9.4; SAS Institute) to perform a stratified blocked randomization [18] with randomly permuted block sizes. Survivors will be randomly allocated to either the waitlist control group or the intervention (ACESO) group, with a 1:1 ratio (N=50). Stratification will be based on breast cancer diagnosis (ie, breast cancer staging and hormone receptor status) and type of breast cancer treatment (radiation, chemotherapy, surgery, and hormone therapy) because they are both associated with the occurrence and severity of treatment-related symptoms and overall quality of life [19-24]. The study navigator will subsequently implement the group allocations and contact patients via an email and follow-up phone call within 3 business days to explain group assignments and assist with the setting up of participant user accounts, usernames, and passwords (for the intervention group). To protect participant confidentiality and privacy, we will encrypt all usernames, passwords, and email addresses used on the website. Participants will be advised to change their assigned password to their own chosen password during the first login. The study navigator who is aware of group assignments will be sequestered from other research assistants and will not administer study surveys or interviews.

### Waitlist Control

The proposed study evaluates an education-based intervention on psychosocial outcomes and will employ a waitlist control condition [25]. Participants assigned to this group will continue to receive usual care and be notified that they are on a waiting list to receive the intervention. The oncology clinic currently provides SCPs to all its patients after treatment completion as part of usual care. However, the paper-based document provides generic information that is not customized for individual patients. Moreover, it does not provide self-tracking of symptoms and quality-of-life indicators affecting breast cancer survivors, and lacks any resources that provide peer support to survivors. After the completion of posttest measures, all participants in the waitlist control group will be provided access and invited to use ACESO.

### Intervention

Participants in this group will also receive the conventional SCP as part of usual care, in addition to access to the ACESO website. After randomization, a nurse-trained study navigator will assist patients with creating and setting up their user accounts, as well as entering their breast cancer diagnosis, treatments, and recommended follow-up schedule data as indicated in their conventional SCP into ACESO. Participants in the intervention group will receive basic training on how to use ACESO and will be familiarized with its features and functions. Participants in this group will use ACESO for (1) logging observed survivor symptoms; (2) built-in self-tracking and charting of treatment-related morbidity (weight, fatigue, depression, anxiety, sexual function, distress, and sleep quality; Table 1 [26-38]); (3) personalized, risk-adapted, and customized educational alerts and tips for symptom management, based on treatment history and self-reported symptoms; (4) email reminders for scheduling SCP-recommended follow-up visits a week prior to a visit due date; (5) monthly reminders and instructions to perform breast self-examinations; (6) an online discussion forum for communicating with other participants in this group about survivorship related topics; and (7) access to a list of evidence-based survivorship resources from NCI and ACS.

**Table 1:** Timing and structure of study outcomes at baseline and 6-month postintervention.

Study outcomes and measurement instruments			Study group	Measure at baseline	Measure at 6 mths	Data source	Cronbach alpha (α)
**Aim 1: Assess ACESO’s feasibility**							
	Patient experience of ACESO: online user experience scale		Intervention	No	Yes	Patient	.88
	Posttest participants’ experience interview		Intervention	No	Yes	Patient	N/A^a^
	Adoption: login frequency, average session duration, days of use, visits by page, discussion forum participation (number of original posts; reply posts)		Intervention	No	Yes	Automated tracking on website	N/A
**Aim 2: Evaluate the extent of ACESO’s impact on breast cancer survivorship**							
	**Primary outcome: treatment-related morbidity**						
		Self-efficacy for self-management (Patient Activation Measure)	Control & intervention	Yes	Yes	Patient	.87
		Patient knowledge (WiSDOM-B^b^)	Control & intervention	Yes	Yes	Patient	N/A
		Perceived peer support	Control & intervention	Yes	Yes	Patient	.90
		Adherence to posttreatment oncologist and primary-care physician visits	Control & intervention	No	Yes	Patient	N/A
	**Secondary Outcome: Treatment-related morbidity**						
		Fatigue (Brief Fatigue Inventory)	Control & intervention	Yes	Yes	Patient	.96
		Depression (CES-D 10^c^)	Control & intervention	Yes	Yes	Patient	.86
		Anxiety (GAD-7^d^)	Control & intervention	Yes	Yes	Patient	.79-.91
		Sexual function (Female Sexual Function Index)	Control & intervention	Yes	Yes	Patient	>.90
		Distress (NCCN^e^ Distress Thermometer)	Control & intervention	Yes	Yes	Patient	.81
		Sleep quality (Pittsburgh Sleep Quality Index)	Control & intervention	Yes	Yes	Patient	.83
		Body weight (participant-owned weighing scale)	Control & intervention	Yes	Yes	Patient	N/A
	**Control Variables**						
		Age, race/ethnicity, education, income, etc.	Control & intervention	Yes	No	Patient	N/A
		Breast cancer diagnosis (staging and hormone receptor status)	Control & intervention	Yes	No	Patient	N/A
		Type of breast cancer treatment	Control & intervention	Yes	No	Patient	N/A

^a^N/A: not applicable.

^b^WiSDOM-B: Wisconsin Survey of Cancer Diagnosis and Management in Breast Cancer.

^c^CES-D-10: 10-item Center for Epidemiological Studies Depression Scale.

^d^GAD-7: 7-item Generalized Anxiety Disorder scale.

^e^NCCN: National Comprehensive Cancer Network.

 All participants will be informed at enrollment that the tool is to support survivor education and for self-management, and that it is not intended to replace the advice of a clinician. The content and presentation of the educational alert messages have been curated by a team of experts, comprising a breast cancer oncologist, a breast cancer nurse practitioner, and 3 breast cancer survivors. All alert messages will include text to consult the primary-care physician if symptoms do not improve or worsen. The online discussion forum will be moderated by a nurse to prevent the sharing of inaccurate and potentially harmful health information, unauthorized use (such as spamming or advertising), hateful conduct, or harassment. However, no research staff will actively participate in conversations on the discussion forum to let conversations among survivors develop organically, and to mitigate the potential of the Hawthorne effect [39]. Participants will be exposed to the intervention for 6 months for the measurement of postintervention outcomes; however, they may continue to use the resource even beyond the study period if they find it beneficial and wish to continue using it.

### Outcome Measures

For the first aim, we will employ a mixed-methods approach [40] to evaluate ACESO’s feasibility based on the survivors’ online user experience and short-term adoption (25/50). Quantitative data on the participants’ online user experience based on their use and perception of ACESO will be collected using a structured survey [41,42]. We will measure online user experience after 6 months of use to measure user perception of ACESO based on 4 dimensions: pragmatic (or utilitarian experience), hedonic (or affective experience), sociability, and usability experience [41,42]. It has been shown that sustained use and adoption for any technological environment depend on whether participants rate these experiences satisfactory or above [41-43]. To measure adoption, we will use automated tracking of logins to ACESO. In addition, we will also track the average time spent for each login session, the number of days of login, the visits for each page of the web application, and discussion group participation (number of original posts and replies to other posts). Qualitative data will be collected by inviting all ACESO users to share their experiences and perceptions of the intervention via a postintervention, in-depth interview conducted on the phone. We will use open-ended questions in combination with probing to gather participant experiences (Multimedia Appendix 3). To ensure participant confidentiality, the research assistants will conduct all interviews in a private, closed room. All interviews will be audiotaped and subsequently transcribed for data analysis by the research assistants.

To measure the primary outcome of self-efficacy for self-management, we will survey all participants at baseline and at 6 months using a structured web-administered questionnaire [27]. To evaluate the survivor’s knowledge of their diagnosis, treatment, and related after-effects, survivor responses to a structured knowledge test [28] will be scored for accuracy by cross-tabulating with the SCP data obtained in the baseline survey. To measure the intervention’s impact on the secondary outcome of treatment-related morbidity, we will utilize a structured web-administered survey using the REDCap survey tool [17] at baseline and at 6 months to evaluate the extent of the intervention’s impact. We will utilize previously developed instruments that have demonstrated high internal consistency and validity in prior studies with cancer patients. Participants will self-report on weight (using participant-owned scales), fatigue [30], depression [32], anxiety [34], sexual function [36], distress [37], and sleep quality [38].

Finally, we will utilize mixed methods to assess perceived peer support among breast cancer survivors. A modification of a structured scale [29] will be used to measure perceived peer support at baseline and postintervention to collect quantitative data. Content analysis [44] will be performed on the online discussion postings made by the participants on ACESO to examine survivor conversations for sources of informational, emotional, and instrumental support by peers. To measure adherence to posttreatment follow-up, we will ask participants to self-report the number of any posttreatment clinical visits made by them at 6 months. We will use their SCP-recommended follow-up schedule data obtained in the baseline survey to compute any missed recommended visits and any additional non-SCP recommended visits made over the last 6 months. We will collect data on both oncology and primary care visits. The structure, timing, and sources for each study outcome are described in Table 1.

### Data Analyses

For the first aim, descriptive statistics (means, percentages, and standard deviations) will be reported for participant demographics and online user experience (for all 4 subscales: pragmatic, hedonic, sociability, and usability). To assess adoption, we will compute the percentage of repeated logins to ACESO (not including the first-time login during the initial set-up); we consider ACESO to be adopted among breast cancer survivors if at least 75% of the users log in at least once after the first-time login. Participants will be classified as adopters or nonadopters, and exact logistic regression will be performed against participant demographics, types of diagnosis, and treatment types. To analyze qualitative data, audio-recordings of participant interviews will be transcribed to text and subsequently coded in NVivo (version 10; QSR International) for thematic analysis [45]. Thematic analysis will allow for the identification of patterns or themes within respondents’ accounts of their experiences with ACESO. Inductive coding [46] of the transcribed recordings will be conducted to allow for the emergence of dominant and frequent themes highlighting any barriers and motivators in using ACESO within the respondents’ narratives. We will use multiple coders to strengthen the validity of the qualitative analysis, who will meet periodically to resolve any coding disagreements.

For the second aim, we will follow the intention-to-treat principle and conduct analysis on data obtained from all enrolled participants, irrespective of the extent of exposure to the intervention. We will report descriptive statistics and confidence intervals for each treatment-related morbidity, patient knowledge, self-efficacy, perceived peer support, and adherence to follow-up visits. Pre-post changes in our primary and secondary outcomes will be compared between the intervention group and the control group using 2-sample *t* tests, chi-square tests, and ANCOVA models, controlled by patients’ breast cancer diagnosis, type of breast cancer treatment, and demographics. To assess follow-up adherence, posttreatment-oncologist and primary-care visits will also be compared between the intervention and the control group using similar models to compare overuse and underuse of follow-up visits between the 2 groups. In addition, time series of observations tracked using ACESO over 6 months (intervention group only) will be plotted and analyzed using repeated measure models for specific change patterns over time.

## Results

**Figure 1:**
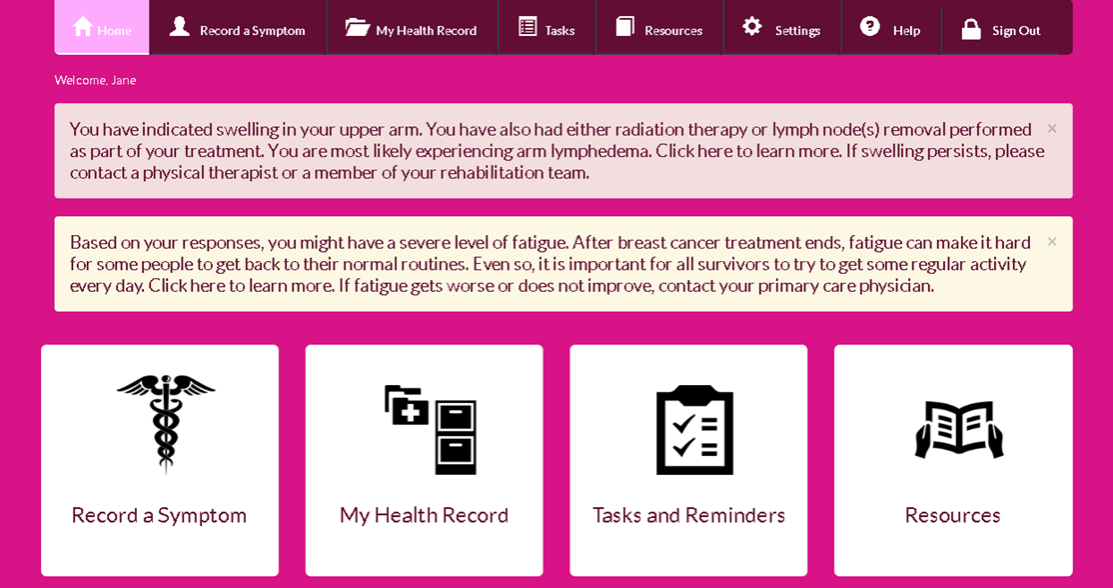
ACESO homepage.

The study will be conducted over a period of 2 years. Approval for the study protocol has been obtained from the Institutional Review Boards of East Carolina University and the University of Wisconsin-Milwaukee. Upon consultation with breast cancer oncologists, survivors, and a nurse, we have developed and refined the online survivorship care plan, ACESO (Figure 1), and subsequently completed an initial usability assessment to identify any issues. In prior usability and acceptance evaluations, survivors indicated high levels of acceptance and interest in the online survivorship plan [14].

We have also established the ACESO Community Advisory Board (ACAB), consisting of breast cancer survivors and health care professionals who will meet biannually and provide community oversight of the proposed research study. The ACAB will make recommendations to the research team to ensure that the study meets its goals in serving the needs of breast cancer survivors and that our recruitment efforts are inclusive and representative of the community. In addition, we have created a National Advisory Committee (NAC) consisting of nationally recognized scientists and advocates in cancer survivorship who will monitor the progress of the study and provide guidance and recommendations to the principal investigators. The NAC will be updated via progress reports before the commencement and achievement of all study milestones. The NAC will convene annually and will be updated on research progress and activities.

## Discussion

ACESO is a one-of-a-kind educational and behavioral intervention that challenges the standard discharge procedure for survivors and seeks to address several inadequacies found in conventional SCPs. The standard discharge process entails a single visit by the survivor after the completion of their initial breast cancer treatment, where they receive the SCP document and an explanation of its contents. This approach is flawed in its assumption of the appropriate timing of the information delivery by the SCPs. The proposed educational intervention delivers time-relevant information and reminders to the survivors at the point of need rather than the point of care; this is especially pertinent given that not all of the information within the SCP is relevant or applicable to individual survivors at the time of discharge, and several treatment-related side effects occur several weeks or more after completing treatment. Moreover, the intervention is radical in the presentation of the content in SCPs. Conventional SCPs include an exhaustive list of common treatment-related side effects experienced by breast cancer survivors as well as corresponding recommendations, which can be overwhelming [47] and may cause a diminished recall over time [48].

The proposed intervention also transforms the static, paper-based SCP into a dynamic SCP, allowing continuous access to the most recent guidelines. As we witness steadily lengthening lifespans of breast cancer survivors, the SCP must shift from being an extant guide of recommendations to a living, organic document that adapts in response to survivors’ changing needs over time [49]. The proposed intervention's dynamic framework makes this possible by providing tailored content in response to survivors’ most current health statuses and care needs. It also ensures that this content is always consistent with the latest guidelines, thus providing constant access to an up-to-date SCP.

ACESO also innovates the format of SCPs by transforming the passive, paper-based SCP into an online, interactive SCP that allows two-way interaction between the survivor and their SCP. In contrast to the paper-based SCP, which employs passive learning based on the reading and retaining of SCP content by survivors, the proposed educational intervention employs active learning to enhance cognition and learning [50].

ACESO incorporates self-regulatory tools and real-time feedback absent in conventional SCPs, such as self-tracking, logging and charting of symptoms and quality of life observations, and tailored alerts (Figure 1). ACESO uses these alerts to provide survivors with real-time feedback based on their observations and to identify specific areas of concern. This combination of self-tracking and real-time feedback has been shown to improve self-efficacy and reactivity for behavior change [51]. Similar patient-centered approaches have previously been shown to improve survivors’ knowledge [52]. Increased levels of self-efficacy and knowledge among survivors should further result in improved physical and psychosocial morbidity [53,54]. ACESO also improves upon the generic, conventional SCP by facilitating personalization and tailoring.

Finally, even though social support, including peer counseling [55], has been shown to be greatly beneficial in improving psychosocial distress, conventional SCPs are devoid of any elements that provide this support to survivors during discharge. ACESO features a discussion group for survivors to interact with peers, develop an online community, and offer informational, emotional, and instrumental peer support, which should prove to be highly beneficial for survivors as they attempt to transition into routine life after treatment.

It is also important to note some of the limitations to the study protocol. Lack of time or interest on the part of prospective participants will be a potential barrier to recruitment. To ensure robust recruitment, we will adopt recruitment procedures previously shown to be successful in our pilot [14] and in other studies [56,57]. In addition, the recruitment window will last a full year to allow for adequate recruitment time. Successful retention of participants for the entire study duration of 6 months is another concern. In addition to offering all participants an incentive (eg, a $50 Amazon gift card) to compensate for their time and effort, we will further split this incentive into 2 installments over the study period (at baseline and 6 months) to promote continued participation. We also acknowledge the likelihood of certain eligible participants not having access to a web-enabled device or the internet. For these participants, we will provide electronic touchscreen tablets with cellular internet connectivity to facilitate participation during the entire course of the study. Certain participants may have limited web literacy skills to meaningfully utilize the online intervention. All participants in the intervention group will receive basic training on how to use ACESO and will be familiarized with its features and functions. We will also provide all participants with clear, structured instructions on operating the website. In addition, the patient navigator will be available for any technical assistance for all participants via the ACESO website, email, or phone (during business hours). We also acknowledge that certain participants will not have access to a weighing scale or will have varying kinds of weighing scales. To improve the internal validity of the data and ensure consistency, we will provide all enrolled and eligible participants with a standard weighing scale to use when self-reporting weight.

By innovatively combining self-monitoring, personalized knowledge delivery, and peer support elements into one comprehensive intervention, the proposed study significantly improves upon standard discharge procedure by equipping survivors with tools that provide the long-term support currently lacking in standard SCPs. Future work should explore the feasibility of directly incorporating patient-generated health data using ACESO into health care providers’ electronic medical record.

The study will help toward significantly advancing current practices in the format, timing, and delivery of SCP content. We expect that this study will reveal variation in posttreatment breast cancer survivorship outcomes among the 2 study groups. Value-laden features within the SCP that allow survivors to track survivor symptoms and quality of life, as well as risk-adapted educational alerts, will have a positive impact on knowledge and self-efficacy for self-management in breast cancer survivors. The results of this pilot trial will inform the feasibility and design of a larger-scale pragmatic trial.

## Appendix

Multimedia Appendix 1 Inclusion enrollment report.

Multimedia Appendix 2 Study timeline.

Multimedia Appendix 3 Postintervention interview questions.

## Abbreviations

ACAB: ACESO Community Advisory Board
ACESO: After-Cancer Education and Support Operations
ACS: American Cancer Society
IOM: Institute of Medicine
NAC: National Advisory Committee
NCI: National Cancer Institute
REDCap: Research Electronic Data Capture
SCP: survivorship care plan
