# Supplementary material for: Exploring Interactive Survivorship Care Plans to Support Breast Cancer Survivors: Protocol for a Randomized Controlled Trial
Source: JMIR Res Protoc. 2020 Dec 4;9(12):e23414. doi: 10.2196/23414 (PMC7748955; doi:10.2196/23414)
Supplement: Multimedia Appendix 1 [file resprot_v9i12e23414_app1.docx]

**PHS Inclusion Enrollment Report**

## *Study Title (must be unique):

**This report format should NOT be used for collecting data from study participants.**

* Delayed Onset Study? Yes No

Interactive Survivorship Care Plans to Support Breast Cancer Survivors using a Personalized Web-Based Educational and Self- Management Tool

***If study is not delayed onset, the following selections are required:***

**Enrollment Type** Planned Cumulative (Actual)

## Using an Existing Dataset or Resource Yes No

**Enrollment Location** Domestic Foreign

## Comments:

## Clinical Trial Yes No NIH-Defined Phase III Clinical Trial Yes No

While the study inclusion criteria does not exclude men, they account for only 1% of the breast cancer survivor population nationally. Therefore, while they will be eligible to participate, due to the difficulty in recruiting prospective male participants, and the limited study period, we do not anticipate any encounter with male breast cancer survivors for this proposed study.

| **Racial Categories** | **Ethnic Categories** | | | | | | | | | |
| --- | --- | --- | --- | --- | --- | --- | --- | --- | --- | --- |
|  | Not Hispanic or Latino | | | Hispanic or Latino | | | Unknown/Not Reported Ethnicity | | | **Total** |
|  | **Female** | **Male** | **Unknown/ Not Reported** | **Female** | **Male** | **Unknown/ Not Reported** | **Female** | **Male** | **Unknown/ Not Reported** |  |
| American Indian/ Alaska Native | 0 | 0 |  | 0 | 0 |  |  |  |  | 0 |
| Asian | 2 | 0 |  | 0 | 0 |  |  |  |  | 2 |
| Native Hawaiian or Other Pacific Islander | 0 | 0 |  | 0 | 0 |  |  |  |  | 0 |
| Black or African American | 12 | 0 |  | 0 | 0 |  |  |  |  | 12 |
| White | 32 | 0 |  | 4 | 0 |  |  |  |  | 36 |
| More than One Race | 0 | 0 |  | 0 | 0 |  |  |  |  | 0 |
| Unknown or Not Reported |  |  |  |  |  |  |  |  |  |  |
| **Total** | 46 | 0 |  | 4 | 0 |  |  |  |  | 50 |

**Report 1 of 1**
