# Supplementary material for: Exploring Interactive Survivorship Care Plans to Support Breast Cancer Survivors: Protocol for a Randomized Controlled Trial
Source: JMIR Res Protoc. 2020 Dec 4;9(12):e23414. doi: 10.2196/23414 (PMC7748955; doi:10.2196/23414)
Supplement: Multimedia Appendix 2 [file resprot_v9i12e23414_app2.docx]

**Study Timeline**

| Activity ↓ / Months → | 1-3 | 4-6 | 7-9 | 10-12 | 13-15 | 16-18 | 19-21 | 22-24 |
| --- | --- | --- | --- | --- | --- | --- | --- | --- |
| Develop study instructional materials | X |  |  |  |  |  |  |  |
| Research Assistants Recruitment & Training | X |  |  |  |  |  |  |  |
| Patient Recruitment |  | X | X | X | X |  |  |  |
| Data Collection |  | X | X | X | X | X | X |  |
| Data Analysis |  |  |  | X | X | X | X |  |
| Dissemination of Results |  |  |  |  | X | X | X | X |
