# Supplementary material for: Exploring Interactive Survivorship Care Plans to Support Breast Cancer Survivors: Protocol for a Randomized Controlled Trial
Source: JMIR Res Protoc. 2020 Dec 4;9(12):e23414. doi: 10.2196/23414 (PMC7748955; doi:10.2196/23414)
Supplement: Multimedia Appendix 3 [file resprot_v9i12e23414_app3.docx]

ACESO Interview Script

**Opening**: I want to thank you for fitting this interview into your schedule.

**Disclosures**: *Setting: Mention recording devices and observers*

This interview is being recorded so that I can write an accurate report – not of who said what, but what was said. Your responses are confidential.

**Permissions**: You are permitted to leave at any time. You may also ask to stop the interview at any time.

1. We will begin by asking you to talk about your breast cancer survivorship experience:
2. After completing your cancer treatment, how well prepared did you feel in terms of taking care of yourself and follow up treatments?
3. How open are you towards using technology to help self-manage your medical condition(s)?
4. How useful did you find the breast cancer survivorship document given to you by your provider after you completed your cancer treatment?
5. Do you think having an app would help/have helped you navigate life after breast cancer much better?
6. Do you think there is a need for more personalized tools (such as apps) to aid breast cancer survivors? Would you use such an app?
7. We will now move on to talk about your experience in using ACESO:
8. Do you have any concerns from using ACESO? (If yes, what are they?)
9. After having used ACESO, can you talk more on the usefulness of such an app?
10. Can you talk about how easy or difficult was it for you to use ACESO?
11. How did ACESO help (or not help) you in managing your breast cancer treatment related symptoms?
12. How much time did you spend using ACESO?
13. Did you share information in ACESO with your doctor, or caregivers? If so, please describe how they used this information.
14. What did you like the most about ACESO?
15. What did you like the least about ACESO?
16. What suggestions would you have to improve ACESO?
17. Do you have any concerns from using ACESO in real life?
18. Do you plan to continue using ACESO if it stays available to you for free?
